# Differences in growth trajectories in breastfed HIV-exposed uninfected and HIV-unexposed infants in Kenya: An observational cohort study

**DOI:** 10.1371/journal.pmed.1004781

**Published:** 2025-10-27

**Authors:** Ruchi Tiwari, Benson O. Singa, Priscah Lihanda, Mame M. Diakhate, Eric Ochola, Lucy Bunyige, Christina Sherry, Barbra A. Richardson, Dalton Wamalwa, Donna M. Denno, Grace C. John-Stewart, Grace M. Aldrovandi, Christine J. McGrath

**Affiliations:** 1 Department of Global Health, University of Washington, Seattle, Washington, United States of America; 2 Center for Clinical Research, Kenya Medical Research Institute, Nairobi, Kenya; 3 Department of Biostatistics, University of Washington, Seattle, Washington, United States of America; 4 Department of Paediatrics and Child Health, University of Nairobi, Nairobi, Kenya; 5 Department of Pediatrics, University of Washington, Seattle, Washington, United States of America; 6 Departments of Epidemiology and Medicine, University of Washington, Seattle, Washington, United States of America; 7 Department of Pediatrics, University of California, Los Angeles, California, United States of America; University of Southampton, UNITED KINGDOM OF GREAT BRITAIN AND NORTHERN IRELAND

## Abstract

**Background:**

Children who are HIV-exposed and uninfected (CHEU) are at increased risk for poor growth compared to children who are HIV-unexposed (CHU). There are limited data on growth among CHEU in the era of preferred dolutegravir-based antiretroviral therapy (ART) for pregnant and breastfeeding women living with HIV (WLWH). We aimed to compare child growth outcomes in the first two years of life between breastfed CHEU and CHU, and to examine maternal HIV factors associated with growth in CHEU.

**Methods and findings:**

We enrolled pregnant women in Kenya and followed them with their child to age 24 months. We measured anthropometry within 7 days of birth, at 3 and 6 weeks, and months 3, 6, 9, 12, 18, and 24. We compared length-for-age Z-scores (LAZ), weight-for-age Z-scores (WAZ), weight-for-length Z-scores (WLZ), head circumference-for-age Z-scores (HCZ), and mid-upper arm circumference-for-age Z-scores (MUAC), and stunting (LAZ < −2), underweight (WAZ < −2), and wasting (WLZ < −2) between groups using linear mixed effects or modified Poisson regression models adjusted for maternal age, education, depression, anemia, household wealth index, time-varying breastfeeding, time-varying food insecurity, parity, and child sex. Among 333 mother-child pairs with at least two child visits (CHEU = 171; CHU = 162), mothers of CHEU were older, less educated, and had lower wealth than mothers of CHU. Birth characteristics were similar between groups, with 9% preterm births and 6% low birthweight. All WLWH were on ART, 89.5% on dolutegravir–lamivudine–tenofovir, 76.6% initiating ART preconception, and 91.2% virally suppressed. The duration of breastfeeding was significantly shorter for CHEU than CHU (median 15 versus 17 months). CHEU had significantly lower LAZ at birth, 18- and 24-months than CHU. In multivariable analysis, growth trajectories for WLZ and HCZ were lower among CHEU than CHU in the first 24 months (interaction *p* = 0.001 and *p* = 0.009, respectively). There was no difference in trajectory in LAZ, WAZ, and MUACZ between groups. By 24 months, 31.5% of CHEU were stunted, 9.3% underweight, and 2.4% wasted, versus 27.2%, 3.2%, and 0.6% of CHU, respectively; only the difference in underweight prevalence was statistically significant. CHEU had a higher risk of being underweight from 9- to 24 months than CHU (adjusted Relative Risk at 24 months, 2.99 [95% CI: 1.08, 8.30]; *p* = 0.034). Growth was associated with maternal education, wealth, and breastfeeding and was lower among male infants. Among CHEU, maternal preconception ART was not associated with growth. Important limitations of this study include the possibility of unmeasured confounding and limited generalizability to contexts with differing prevalence of malnutrition, access to and uptake of ART, or breastfeeding practices.

**Conclusions:**

Despite breastfeeding and optimal maternal dolutegravir-based ART, CHEU experienced growth deficits compared to CHU in the first two years of life. Continued monitoring of the expanding CHEU population is essential in the context of rapidly evolving guidelines and policies to optimize their health and to identify and prevent future health disparities and disease risks.

## Author summary

**Why was this study done?:**

- Children who are HIV-exposed and uninfected (CHEU) are at increased risk for poor growth compared to children who are HIV-unexposed (CHU).
- In 2016, WHO extended the continued breastfeeding duration guidance for lactating women living with HIV (WLWH) adherent to antiretroviral therapy (ART) from 12 months to at least 24 months for those residing in countries that promote and support breastfeeding with ART.
- The WHO now recommends dolutegravir (DTG)-based ART as the preferred first-line treatment for pregnant WLWH. However, few studies compare growth between CHEU and CHU in the context of updated ART guidelines and safer, sustained breastfeeding practices among WLWH.

**What did the researchers do and find?:**

- We compared growth trajectories of breastfed Kenyan CHEU and CHU in the first two years of life. This is one of the few studies conducted in the context of universal ART and sustained breastfeeding, where most WLWH (90%) were on the WHO preferred first-line ART, DTG–lamivudine–tenofovir, during pregnancy.
- Our findings show that CHEU had significantly slower weight-for-length and head circumference-for-age growth in the first 2 years, and were more likely to be underweight from 9 to 24 months than CHU. Stunting prevalence increased with age in this population, and by 24 months, nearly one-third of all children were stunted.

**What do these findings mean?:**

- Our study highlights growth deficits, particularly poorer weight gain and slower weight-for-length growth among breastfed CHEU. These findings may reflect lasting metabolic or epigenetic effects from in utero exposure to ART. They underscore the need for further mechanistic research to elucidate biological pathways underlying poor growth outcomes among CHEU and to help guide the selection of the safest ARV regimens during pregnancy.
- Continued growth monitoring is critical as HIV and ART guidelines evolve.
- The main limitation of our study is its limited representativeness of the broader CHEU population, due to differences in access to ART during pregnancy and postpartum, duration of exclusive and sustained breastfeeding, and prevalence of child malnutrition. Consequently, additional population-based studies in diverse settings are needed to validate these findings.

## Introduction

An estimated 16 million children globally are HIV-exposed in utero and uninfected (CHEU), with 1.2 million CHEU born annually [1]. In high HIV prevalence settings of sub-Saharan Africa, as many as 1 in 5 children are born to women living with HIV (WLWH). [2,3] CHEU have been shown to have increased risk of growth impairment, infectious morbidity, and mortality compared to children who are HIV-unexposed (CHU) [4–7]. The mechanisms responsible for poor growth in CHEU are not well understood and may depend in part on *in utero* exposure to HIV and antiretroviral therapy (ART), although results are inconsistent.

The World Health Organization (WHO) recommends ART for all pregnant and breastfeeding WLWH for the prevention of vertical transmission of HIV and the health of the mother, although some studies report that in utero exposure to ART negatively impacts child growth [8–13]. In 2018, Kenya was one of the first countries in sub-Saharan Africa to implement the guideline change to include dolutegravir (DTG) as a component of first-line ART (tenofovir, lamivudine, DTG), which included women of reproductive potential, pregnant, and breastfeeding women [14]. In 2016, WHO extended the continued breastfeeding duration guidance for lactating WLWH adherent to ART therapy and residing in countries that promote and support breastfeeding with ART from 12 months to at least 24 months [15]. Recent studies from the current era of maternal use of early and effective suppressive combination ART and safer and sustained breastfeeding by WLWH continue to report suboptimal growth and a higher risk of growth faltering among CHEU [16]. However, there is a lack of comparative studies on short- and longer-term growth outcomes among breastfed CHEU following in utero exposure to the currently recommended first-line ART regimens for HIV, particularly those containing DTG.

Several factors potentially contribute to poor growth among CHEU. Evidence is mixed on the extent to which in utero exposure to maternal ART, exposure through breastfeeding, or prophylactic antiretroviral (ARV) use is directly associated with child growth [17–20]. Nevertheless, considering optimized ART and breastfeeding among WLWH on ART, it is critical to continue to monitor growth trajectories of CHEU. Optimal maternal ART results in maternal viral suppression that protects maternal health, reduces the risk of vertical HIV transmission and allows CHEU to safely benefit from breastfeeding [21]. Emerging evidence among adults suggests DTG-based ART is associated with significant weight gain, increasing body mass index (BMI), and in the long-term may contribute to other metabolic complications [22,23]. Pregnant women on DTG-based ART may experience changes to maternal physiology such as greater weight gain during pregnancy [24] and hyperglycemia [25], that may affect fetal development and influence metabolic health in the child. However, it is unclear whether DTG-related alterations in maternal metabolic health influence child growth outcomes. We hypothesized that breastfed CHEU exposed to DTG-based ART would exhibit growth trajectories comparable to or exceeding those of CHU in the same community. The objective of this study was to compare child growth outcomes in the first two years of life between breastfed CHEU and CHU, and to examine maternal HIV-related factors associated with growth in CHEU in the context of current treatment and breastfeeding guidelines.

## Methods

### Study design, site, and population

The Tunza Mwana prospective cohort study enrolled pregnant women living with and without HIV attending antenatal clinic visits and followed the mother-child dyads to 24 months postpartum. Pregnant women were recruited from Migori County Referral Hospital and St. Joseph’s Mission Hospital in western Kenya between November 17, 2020, and November 29, 2021. Both are level-four primary care hospitals in Migori County, Kenya, providing a range of health services, including antenatal, delivery, and postpartum care, and serving diverse populations. Migori County is a rural, agriculturally productive county in western Kenya with 15% prevalence of stunting [26] in children under 5 years of age and 16% HIV prevalence among women [27]. Eligibility criteria included: between 28 and 42 weeks’ gestation, aged 18–40 years, planned to primarily breastfeed their infants for at least 6 months, remain in the catchment area for 24 months, and were willing to attend study visits with their child, and utilize HIV services if WLWH. HIV results among women self-reporting HIV–positive status were verified by the Maternal and Child Health (MCH) Booklet. Women were excluded if they were not willing to disclose their HIV status and/or have an HIV test at screening or were diagnosed with hypertension, pre-eclampsia, or other serious medical condition that was likely to result in early inducement or scheduled Cesarean section. Gestational age for screening was estimated using last menstrual period.

The study was approved by the Kenya Medical Research Institute Scientific and Ethics Review Unit (0140/3940) and the University of Washington Institutional Review Board (STUDY00007708). All women provided written informed consent for themselves and their child to participate in the study.

### Inclusivity in global research

Additional information regarding the ethical, cultural, and scientific considerations specific to inclusivity in global research is included in the Supporting Information (S1 Inclusivity Checklist).

### Procedures

At enrollment, trained study staff ascertained participant sociodemographic characteristics, dietary and household food insecurity status, obstetric and medical history, including ART regimen and start date, cotrimoxazole use. Blood was drawn for HIV testing in women not known to be living with HIV. Maternal weight, height, and mid-upper arm circumference (MUAC) were measured, and the presence of depressive symptoms was assessed using the Patient Health Questionnaire-9 (PHQ-9) at all study visits. Medical records were abstracted for relevant obstetric and health information.

Mother-child pairs returned for study visits within 7 days of birth, at 3 and 6 weeks of age, and at 3, 6, 9, 12, 18, and 24 months of age. Delivery and birth data were abstracted from medical records. Children’s length, weight, head circumference, and MUAC were measured at each follow-up visit using standardized procedures. Recumbent length was measured to the nearest 0.1 cm with a ShorrBoard by two study staff (measurer and assistant). Weight was recorded to the nearest 0.1 kg using a digital ADE scale with child undressed. MUAC and head circumference were measured to the nearest 0.1 cm using standard MUAC and infant head circumference tapes. The average of two measurements was used unless discrepancies exceeded 0.2 cm for length, 0.1 kg for weight, 0.5 cm for MUAC, or 0.2 cm for head circumference, in which case a third measurement was taken, and the average of the three measurements was used. Digital scales were calibrated daily. Study staff collected information on maternal and child illness, hospitalization, and medication use, including antibiotics. Among CHEU, additional data was collected on receipt of ARV prophylaxis from birth and cotrimoxazole prophylaxis from age 6 weeks. Household food security status and child feeding practices, including timing of breastfeeding initiation, date of last breastfeeding, and date of introduction of other liquids and/or foods were assessed by maternal report at each visit. Breastfeeding counseling and guidance on infant nutrition was provided to mothers at each study visit.

### Inclusion criteria for growth analysis

The present analysis included children with at least two separate weight, length, head circumference, or MUAC measurements from birth to 24 months of age. In the case of twins (*n* = 1), only the first-born child was included in this analysis. The last follow-up visit for the final participant occurred on February 6, 2024. This study is reported as per the Strengthening the Reporting of Observational Studies in Epidemiology (STROBE) guideline (S1 STROBE Checklist).

### Child HIV exposure status

Children’s HIV exposure status was based on the mother’s HIV status at enrollment. Children born to WLWH underwent HIV PCR testing at age 6 weeks, and 6 and 12 months and HIV antibody testing at age 18 months per Kenya National Guidelines [28]. Women who tested negative for HIV in pregnancy were retested at 6 weeks after delivery and thereafter every 6 months until cessation of breastfeeding per national guidelines. All enrolled women and children remained in the study and were followed up even if their HIV status changed during the follow-up period. Children born to WLWH at enrollment were considered CHEU unless the child tested HIV–positive during follow-up. Children born to HIV–negative women at enrollment were classified as CHU unless the mother tested HIV–positive in the postpartum period. One CHU whose mother tested HIV–positive at 1–7 days of postpartum time point was excluded from the present growth analysis. Data were excluded after the last known HIV–negative time point for two children HIV exposed who tested HIV–positive at 6 and 9 months (both of whom had a negative HIV PCR test result at 6 weeks of age).

### Covariates

At enrollment, maternal weight, height, and MUAC were measured by trained study staff with at least two readings for each measure. Maternal nutritional status based on MUAC was categorized as follows: undernourished (<23 cm), normal (23–30.5 cm), and overweight/obese (>30.5 cm) [29,30]. Household food insecurity status was calculated based on the Household Food Insecurity Access Scale (HFIAS) [31]. Information on household assets (any livestock, bank account, electricity, radio, tv, refrigerator, bike, scooter, smartphone, standard phone, improved drinking water [32], improved cooking [33], improved floor, improved roof, improved wall, improved sanitation [32,34]) was used to calculate wealth index by principal component analysis after imputing missing values using the mice: Multivariate Imputation by Chained Equations in R. We used the first principal component to represent the household wealth index. A single imputed dataset for missing wealth-index related variables mentioned above was utilized. Depression was categorized based on the scores from PHQ-9 using the following categories: no depression (score 0–4), mild depression (score 5–9), moderate or severe depression (score ≥10). Maternal hemoglobin level at enrollment was abstracted from the mother’s MCH booklet. Gestational age at birth was abstracted from the delivery records or MCH booklet or reported by mother at the delivery visit (1–7 days postpartum). If the mother missed the delivery visit (1–7 days postpartum) and gestational age at delivery was not available in the delivery records or MCH booklet, it was calculated from self-reported date of last menstrual period and date of delivery, or gestational age at screening and the number of weeks between screening and delivery.

### Outcomes

An average of two readings of length, weight, head circumference, and MUAC was collected from children at each time point. If the difference between the first two measurements was greater than a set value (length 0.5 cm, weight 0.10 kg, head circumference 0.5 cm, MUAC 0.5 cm), a third measurement was obtained, and average of three readings was used to determine the final value. Length-for-age Z-scores (LAZ), weight-for-age Z-scores (WAZ), weight-for-length Z-scores (WLZ), head circumference-for-age (HCZ), and MUAC Z-scores were calculated using the WHO Child Growth Standards Anthro package (version 1.0.0) in R. If a length or head circumference measurement was 0.5 cm or more lower than a measurement from a previous visit, the measurement was excluded. Similarly, any Z-score value beyond a pre-defined WHO range or flag limit (−6 < LAZ > +6, −6 < WAZ > +5, −5 < WLZ > +5, −5 < HCZ > +5, −5 < MUACZ > +5) was compared to prior and subsequent measurements and if considered an implausible or potentially incorrect value, it was removed from the analysis. Stunting (LAZ < −2), wasting (WLZ < −2, underweight (WAZ < −2), overweight (WAZ > 2), microcephaly (HCZ < −2), and macrocephaly (HCZ > 2) status was calculated at each visit.

### Sample size

The Tunza Mwana study aimed to evaluate the association between maternal HIV, breastmilk composition, and the infant gut microbiome, and identify breast milk-mediated pathways associated with morbidity and linear growth in CHEU. Based on 5% mother-to-child HIV transmission rate in western Kenya and including 20% attrition in each group, the required sample size was estimated at 175 CHEU and 175 CHU. With 80% power and assuming a standard deviation (SD) of 1.0 in mean LAZ, the minimum detectable difference in mean LAZ between groups was 0.42 SD using a two-sided test with *α* = 0.05.

### Statistical analysis

Analyses were conducted following a predetermined approach, although no formal prespecified analysis plan was established. At no point were data-driven approaches used. Sociodemographic, pregnancy, and infant-related characteristics of the mother-child pairs were examined overall and by the HIV exposure status at enrollment. Median and interquartile range (IQR) or mean and SD were calculated for continuous variables and the percent of total for categorical variables. Missing values for infant length at 1–7 days after birth were imputed using MICE: Multivariate Imputation by Chained Equations in R [35] and where available, missing values of infant weight at 1–7 days after birth were abstracted from the delivery record, and if unavailable, were imputed. We imputed data 10 times and compared the observed and imputed data to assess the plausibility of the imputations. Variables used for imputation were maternal HIV status, education, height, child sex, and available measures of length, weight, MUAC, head circumference at follow-up visits. We utilized the first imputed dataset to obtain the imputed values for weight and length missed at the delivery visit. All outcomes were summarized separately for CHEU and CHU, by follow-up time points. For each visit, the mean difference in Z-scores comparing CHEU and CHU was calculated. In addition, the difference in the proportion of stunted, wasted, and underweight between the two groups was determined using Pearson’s Chi-squared tests. The growth trajectory for continuous measures of LAZ, WAZ, WLZ, HCZ, and MUACZ between follow-up visits was estimated using adjusted linear mixed models with random effects for intercept. The adjusted mixed effect models included the exposure group, visit time point representing age of the child as factor (1–7 days, 3 and 6 weeks, 3, 6, 9, 12, 18, and 24 months) and interaction terms between the exposure and the time points, along with the following adjustment variables: maternal age, any breastfeeding at each visit, education, depression, anemia, wealth index, food insecurity at each visit, parity, and child sex. These variables were selected *a priori*, and a directed acyclic graph was drawn to examine the relationship among covariates and to identify the minimum set of variables (S1 Table). The global *p*-value for the interaction between time (study visit) and exposure groups was used to test whether the growth trajectory differed between CHEU and CHU, while adjusting for other covariates. The same adjusted mixed effects model was used to further explore cofactors that influenced child growth outcomes. The risk of stunting, wasting, underweight, overweight, macrocephaly and microcephaly at each follow-up visit associated with HIV exposure status was examined using unadjusted and adjusted Poisson regression models with robust standard errors. Finally, linear mixed effects and logistic regression models were conducted among CHEU to examine whether the outcomes differed by timing of maternal ART initiation (before versus during pregnancy). Separate adjusted regression models including maternal ART initiation (before versus during pregnancy) in addition to the other covariates listed above were performed. To evaluate if the findings differed by maternal ART regimen, all above analyses were repeated among women on DTG-based ART, where we compared outcomes between CHEU exposed to DTG-based ART and CHU. In all analyses, the p-value was two-sided and statistical significance was calculated at *α* = 0.05. Analyses were conducted using R studio software (R Version 4.1.1 [2021-08-10]).

## Results

Among the 350 pregnant women enrolled in the Tunza Mwana cohort, 333 mother-child pairs attended at least two follow-up visits and were included in the analysis (S1 Fig). Of the 333 mother-child pairs, 165 CHEU and 158 CHU completed 24-month follow-up with a mean of 8.8 visits per child (Mean [SD]: 8.8 [0.8] CHEU, 8.7 [0.7] CHU). At enrollment, pregnant WLWH were older, less educated, and in the lower two wealth quintiles than pregnant women without HIV (Table 1). A greater proportion of WLWH were anemic in pregnancy and reported household food insecurity compared to their HIV-uninfected counterparts. All WLWH were on ART in pregnancy with 89.5% on the WHO preferred first-line regimen of DTG–lamivudine–tenofovir and 76.6% initiated ART preconception. Preterm and low birth weight prevalences were similar among CHEU and CHU (9.3% preterm births and 6.0% low birthweight overall). CHEU were less likely to be first-born than CHU (9.2% versus 33.3%). The average number of children living in the house was slightly higher for CHEU than CHU household (Mean [SD]: 2.4 [1.5] CHEU, 1.4 [1.5] CHU, *p* < 0.001). CHEU breastfed for a shorter duration than CHU (median 15 versus 17 months, *p* < 0.01). A greater proportion of CHEU were exclusively breastfed for 6 months, yet fewer CHEU continued to breastfeed at 12 months and beyond compared to CHU.

**Table 1:** Characteristics of pregnant women at enrollment and their children overall and by maternal HIV status.

	*n* (%) or median (IQR)			*p*-value^a^
	Women living with HIV and CHEU (*n* = 171)	Women HIV-uninfected and CHU (*n* = 162)	All (*n* = 333)	
***Site***				0.123
Migori County Referral Hospital, Migori	103 (60.2%)	83 (51.2%)	186 (55.9%)	
St. Joseph Mission Hospital, Migori	68 (39.8%)	79 (48.8%)	147 (44.1%)	
***Maternal household and clinical characteristics***				
Age				
≤ 25 years	38 (22.2%)	86 (53.1%)	124 (37.2%)	<0.001
26–35 years	97 (56.7%)	61 (37.7%)	158 (47.4%)	
≥ 35 years	36 (21.1%)	15 (9.3%)	51 (15.3%)	
Maternal Education				<0.001
No education/less than primary	52 (30.4%)	19 (11.7%)	71 (21.3%)	
Completed primary education	45 (26.3%)	34 (21.0%)	79 (23.7%)	
Not completed secondary education	36 (21.1%)	28 (17.3%)	64 (19.2%)	
Completed secondary education or above	38 (22.2%)	81 (50%)	119 (35.7%)	
Married	143 (83.6%)	139 (85.8%)	282 (84.7%)	0.690
Food insecurity				0.026
Secure	39 (22.8%)	58 (35.8%)	97 (29.1%)	
Mild	18 (10.5%)	21 (13.0%)	39 (11.7%)	
Moderate	67 (39.2%)	43 (26.5%)	110 (33.0%)	
Severe	44 (25.7%)	40 (24.7%)	84 (25.2%)	
Wealth index				0.019
Quintile 1	41 (24.0%)	27 (16.7%)	68 (20.4%)	
Quintile 2	41 (24.0%)	25 (15.4%)	66 (19.8%)	
Quintile 3	35 (20.5%)	31 (19.1%)	66 (19.8%)	
Quintile 4	29 (17.0%)	39 (24.1%)	68 (20.4%)	
Quintile 5	25 (14.6%)	40 (24.7%)	65 (19.5%)	
Depressive symptom screening				0.324
No to minimal depressive symptoms	114 (66.7%)	121 (74.7%)	235 (70.6%)	
Mild depressive symptoms	51 (29.8%)	36 (22.2%)	87 (26.1%)	
Moderate or severe depressive symptoms	6 (3.5%)	5 (3.1%)	11 (3.3%)	
Height (cm)	162 (158, 166)	161 (157, 166)	162 (157, 166)	0.079
Overweight/obese (MUAC >30.5 cm)	32 (18.7%)	30 (18.5%)	62 (18.6%)	0.720
Gestational age, weeks	31.5 (29.5.0, 35.8)	34.0 (31.8, 36.5)	32.5 (31.0, 36.0)	0.389
Anemia (<11.0 g/dL)	68 (39.8%)	33 (20.4%)	101 (30.3%)	<0.001
Multiparous	157 (91.8%)	108 (66.7%)	265 (79.6%)	<0.001
***Maternal HIV characteristics***				
ART initiation before pregnancy	131 (76.6%)			
Time since ART initiation				
<6 months	28 (16.4%)			
6–12 months	13 (7.6%)			
12–24 months	130 (76.0%)			
ART regimen at enrollment				
Atazanavir/ritonavir, lamivudine, zidovudine	5 (2.9%)			
Atazanavir/ritonavir, lamivudine, tenofovir	8 (4.7%)			
Atazanavir/ritonavir, lamivudine, abacavir	1 (0.6%)			
Dolutegravir, lamivudine, zidovudine	1 (0.6%)			
Dolutegravir, lamivudine, tenofovir	153 (89.5%)			
Efavirenz, lamivudine, tenofovir	3 (1.8%)			
Cotrimoxazole prophylaxis	110 (64.3%)			
HIV viral suppression (<1,000 copies/mL)	156 (91.2%)			
***Birth and infant characteristics***				
Female	81 (47.4%)	75 (46.3%)	156 (46.8%)	0.931
Gestational age at delivery, weeks	39.0 (38.0, 40.0)	39.2 (38.0, 40.0)	39.1 (38.0, 40.0)	0.093
Cesarean delivery	20 (11.7%)	19 (17.9%)	49 (14.7%)	0.147
Preterm birth (<37 weeks)	16 (9.4%)	15 (9.3%)	31 (9.3%)	1.000
Birth weight within 7 days of birth, kg^b^	3.2 (2.9, 3.6)	3.3 (2.9, 3.6)	3.2 (2.9, 3.6)	0.339
SGA^c^	20 (11.7%)	9 (5.6%)	29 (8.7%)	0.071
Low birth weight (<2.5 kg)^d^	10 (5.8%)	10 (6.2%)	20 (6.0%)	1.000
Length within 7 days of birth, cm^e^	49.3 (48.3, 50.9)	49.9 (48.4, 51.2)	49.6 (48.3, 51.0)	0.038
ARV prophylaxis from birth^f^				
Nevirapine plus zidovudine	152 (88.9%)			
Zidovudine alone	4 (2.3%)			
Nevirapine alone	15 (8.8%)			
***Breastfeeding***				
Exclusive breastfeeding for 6 months	100 (58.5%)	83 (51.2%)	183 (55.0%)	0.180
Breastfeeding at 12 months	144 (84.2%)	147 (90.7%)	291 (87.4%)	<0.001

Number of missing values: food insecurity (CHU = 3), anemia (CHU = 4, CHEU = 18), parity (CHEU = 2), cesarean delivery (CHEU = 3, CHU = 3), birthweight (CHU = 1, CHEU = 1), SGA (CHEU = 14, CHU = 13), low birth weight (CHU = 1, CHEU = 1), length within 7 days (CHU = 1, CHEU = 1), ARV prophylaxis from birth (CHEU = 29), ever on cotrimoxazole (CHEU = 1), exclusive breastfeeding for 6 months (CHEU = 2), breastfeeding at 12 months (CHU = 2, CHEU = 3).

^a^*p*-values are from Kruskal–Wallis test for median and chi-squared test or Fisher’s exact test for categorical variables.

^b^Weight values are imputed for 1 CHEU and 4 CHU who missed the follow-up visit within 7 days of birth and weight was not recorded in their delivery record.

^c^SGA is based on weight on the day of birth.

^d^Low birth weight is based on birth weight obtained within 7 days of birth.

^e^Length imputed for 5 CHEU and 16 CHU who missed the follow-up visit within 7 days of birth.

^f^Infant ARV prophylaxis includes the first reported regimen.

Abbreviations: IQR, interquartile range; CHEU, children HIV exposed uninfected; CHU, children HIV unexposed uninfected; MUAC, mid-upper arm circumference; ARV, antiretroviral; ART, antiretroviral therapy; SGA, small for gestational age.

**Fig 1:**
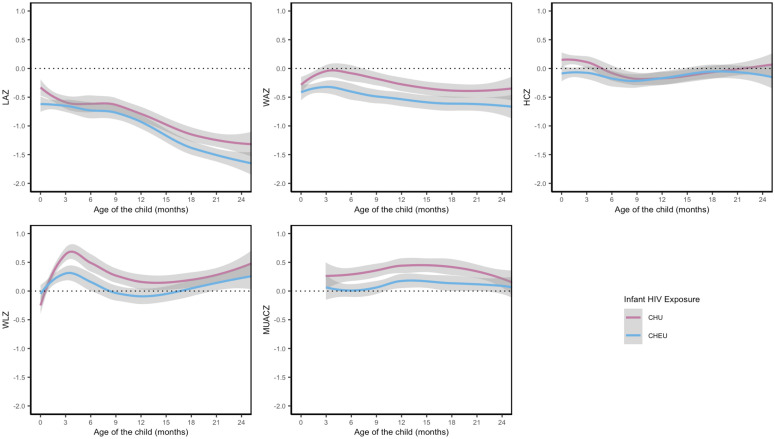
Two-year growth trajectory of children HIV exposed uninfected (CHEU) and HIV-uninfected (CHU) using LOESS regression. The lines on each graph were fit using LOESS regression and the shaded area represents 95% confidence interval. The child’s age at each follow-up visit was calculated based on the recorded date of birth and the date of anthropometric assessment. CHEU, children HIV exposed uninfected; CHU, children HIV unexposed uninfected; LAZ, length-for-age Z-score; WAZ, weight-for-age Z-score; WLZ, weight-for-length Z-score; HCZ, head-circumference-for-age Z-score; MUAC, mid-upper arm circumference; MUACZ, MUAC-for-age Z-score.

**Fig 2:**
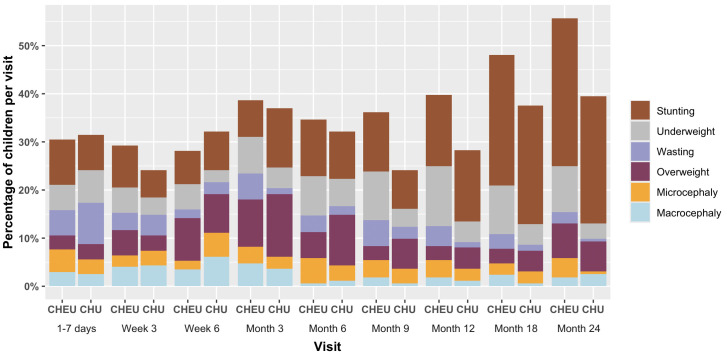
Growth faltering^a^ in the first 24 months of life by study visit^b^ and child’s HIV exposure. CHEU: Children HIV exposed uninfected; CHU: Children HIV unexposed. ^a^Growth faltering included overlapping categories of stunting (LAZ < −2), wasting (WLZ < −2, underweight (WAZ < −2), overweight (WAZ > 2), microcephaly (HCZ < −2), and microcephaly (HCZ > 2). ^b^Total number of children included at each follow-up visit: 1-7 days of birth: 333 (CHEU: 171, CHU: 162); Week 3 visit: 315 (CHEU: 166, CHU: 149); Week 6 visit: 328 (CHEU: 169, CHU: 159); Month 3 visit: 329 (CHEU: 168, CHU: 161); Month 6 visit: 328 (CHEU: 167, CHU: 161); Month 9 visit: 326 (CHEU: 166, CHU: 160); Month 12 visit: 326 (CHEU: 166, CHU: 160); Month 18 visit: 326 (CHEU: 165, CHU: 161); Month 24 visit: 323 (CHEU: 165, CHU: 158).

In the first seven days of life, mean LAZ was significantly lower among CHEU (−0.67 [SD 1.12]) than CHU (−0.39 [SD 1.15]) (Table 2). Mean LAZ remained lower for CHEU than CHU throughout follow-up with significantly lower LAZ at 18- and 24-months (Table 2 and Fig 1). Mean LAZ between 9- and 18-months declined rapidly in both groups with a stabilization at 24-months (Table 2 and Fig 1). In regression analysis adjusted for maternal age, any breastfeeding at each visit, education, depression, anemia, wealth index, food insecurity at each visit, parity, and child sex, the overall trajectory for LAZ was similar between CHEU and CHU throughout 24-month follow-up (interaction *p* = 0.224). By 24 months, 31.5% of CHEU were stunted (LAZ < −2) compared to 27.2% of CHU with no difference between the groups throughout follow-up (Fig 2 and S2 Table).

**Table 2:** Anthropometric measurements among children HIV exposed uninfected and children HIV uninfected by study visit^a^.

Study visit	Mean (SD)								
	Length (cm)	Wgt (kg)	Head cir. (cm)	MUAC (cm)	LAZ	WAZ	WLZ	HCZ	MUACZ
***Children HIV exposed uninfected (CHEU)***									
1–7 days of birth	49.4 (2.2)	3.2 (0.5)	34.8 (1.5)	NA	−0.7 (1.1)*	−0.5 (1.0)	−0.2 (1.2)	−0.1 (1.2)	NA
Week 3	52.1 (2.3)	3.9 (0.6)	36.3 (1.5)	NA	−0.6 (1.1)*	−0.3 (1.1)	0.2 (1.2)	−0.1 (1.2)	NA
Week 6	54.7 (2.2)	4.7 (0.7)	37.7 (1.4)	NA	−0.6 (1.1)	−0.3 (1.1)	0.4 (1.2)	−0.1 (1.1)*	NA
Month 3	59.5 (2.4)	5.9 (0.9)	40.1 (1.5)	13.4 (1.2)	−0.6 (1.1)	−0.3 (1.2)*	0.3 (1.3)**	0.2 (1.1)	0.1 (1.1)
Month 6	65.0 (2.6)	7.3 (0.9)	42.5 (1.5)	14.1 (1.2)	−0.7 (1.1)	−0.4 (1.2)*	0.2 (1.3)*	−0.2 (1.1)	−0.0 (1.1)*
Month 9	69.4 (2.6)	8.2 (1.1)	44.5 (1.5)	14.4 (1.1)	−0.7 (1.1)	−0.5 (1.1)*	−0.1 (1.2)*	−0.2 (1.1)	0.1 (0.9)**
Month 12	72.5 (2.6)	8.9 (1.2)	45.3 (1.5)	14.7 (1.1)	−0.9 (1.1)	−0.5 (1.2)	−0.1 (1.3)	−0.1 (1.1)	0.2 (0.9)*
Month 18	77.3 (3.2)	9.9 (1.2)	46.8 (1.5)	14.9 (1.1)	−1.5 (1.1)*	−0.7 (1.1)*	0.1 (1.2)	0.0 (1.0)	0.1 (0.9)**
Month 24	82.0 (3.4)	11.2 (1.4)	47.6 (1.5)	15.2 (1.1)	−1.5 (1.1)*	−0.5 (1.1)	0.3 (1.3)	−0.1 (1.0)	0.1 (0.9)
***Children HIV unexposed (CHU)***									
1–7 days of birth	49.9 (2.2)	3.3 (0.5)	34.9 (1.3)	NA	−0.4 (1.2)*	−0.4 (1.0)	−0.4 (1.3)	0.1 (1.1)	NA
Week 3	52.6 (2.1)	4.0 (0.6)	36.6 (1.4)	NA	−0.4 (1.1)*	−0.2 (1.0)	0.1 (1.2)	0.2 (1.2)	NA
Week 6	54.9 (2.2)	4.8 (0.6)	38.1 (1.4)	NA	−0.5 (1.1)	−0.1 (0.9)	0.6 (1.1)	0.2 (1.1)*	NA
Month 3	59.5 (2.2)	6.2 (0.8)	40.3 (1.3)	13.7 (1.1)	−0.6 (1.0)	−0.0 (1.0)*	0.7 (1.1)**	0.2 (1.0)	0.3 (1.1)
Month 6	65.6 (2.6)	7.6 (1.1)	42.7 (1.3)	14.4 (1.2)	−0.6 (1.1)	−0.1 (1.2)*	0.4 (1.3)*	−0.1 (0.9)	0.3 (1.1)*
Month 9	69.7 (2.5)	8.5 (1.2)	44.2 (1.3)	14.8 (1.2)	−0.6 (1.1)	−0.2 (1.1)*	0.2 (1.2)*	−0.2 (0.9)	0.4 (0.9)**
Month 12	72.9 (2.7)	9.1 (1.2)	45.4 (1.3)	15.0 (1.2)	−0.9 (1.1)	−0.3 (1.1)	0.2 (1.1)	−0.2 (0.9)	0.5 (0.9)*
Month 18	78.2 (3.3)	10.2 (1.3)	46.8 (1.3)	15.2 (1.2)	−1.2 (1.2)*	−0.4 (1.1)*	0.2 (1.1)	0.1 (0.9)	0.4 (0.9)**
Month 24	82.9 (3.7)	11.4 (1.5)	47.8 (1.3)	15.4 (1.2)	−1.3 (1.2)*	−0.4 (1.0)	0.4 (0.9)	0.1 (0.9)	0.2 (0.9)

*p-value <0.05 **p-value <0.01 from *t* tes*t* (comparison using *t* test was only performed for LAZ, WAZ, WLZ, HCZ, and MUACZ).

^a^Total number of children included at each follow-up visits: 1–7 days of birth: 333 (CHEU: 171, CHU: 162); Week 3 visit: 315 (CHEU: 166, CHU: 149); Week 6 visit: 328 (CHEU: 169, CHU: 159); Month 3 visit: 329 (CHEU: 168, CHU: 161); Month 6 visit: 328 (CHEU: 167, CHU: 161); Month 9 visit: 326 (CHEU: 166, CHU: 160); Month 12 visit: 326 (CHEU: 166, CHU: 160); Month 18 visit: 326 (CHEU: 165, CHU: 161); Month 24 visit: 323 (CHEU: 165, CHU: 158).

Abbreviations: CHEU, children HIV exposed uninfected; CHU, children HIV unexposed uninfected; SD, standard deviation; MUAC, mid-upper arm circumference; LAZ, length-for-age Z-score; WAZ, weight-for-age Z-score; WLZ, weight-for-length Z-score; HCZ, head-circumference-for-age Z-score; MUACZ, MUAC-for-age Z-score; NA, not applicable.

Mean WAZ increased slightly following birth with gradual declines from week-6 for CHEU and month-3 for CHU through 18 months, and mean WAZ remained below the population average of the WHO referrant population (Z-score = 0) throughout follow-up for both groups (Table 2 and Fig 1). Between 18- and 24-months, WAZ increased slightly in both groups. CHEU had significantly lower WAZ than CHU at 3-, 6-, 9- and 18-months (Table 2). Results of adjusted regression models show that the mean WAZ trajectory in the first 24-months of life was not significantly different between CHEU and CHU (interaction *p* = 0.387), although CHEU had lower mean WAZ at all study visits. The proportion of children who were underweight (WAZ < −2) was higher at all visits for CHEU than CHU (Fig 2 and S2 Table). In adjusted analysis, CHEU were significantly more likely to be underweight than CHU at month 9, 12, 18, and 24 (adjusted RR [aRR]: 3.18 [95% CI: 1.33, 7.58], 3.62 [95% CI: 1.75, 7.48], 3.58 [95% CI: 1.64, 7.83], and 2.99 [95% CI: 1.08, 8.30], respectively for month 9, 12, 18, and 24).

**Fig 3:**
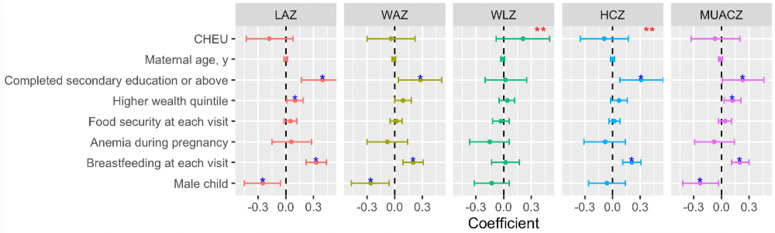
Coefficients of selected variables from multivariable mixed effects linear regression model. β (95% CI) are difference in Z-scores between the groups specified obtained from mixed-effects regression model with an interaction term between time (study visit) and HIV exposure status and the following: maternal age (years), currently breastfeeding at each follow-up visit (yes/no), education (Secondary and above vs. Primary or below), depression (yes/no), anemia (yes/no), wealth index (grouped linear variable – 0 = lowest quintile, 1 = quintile 2, 2 = quintile 3, 3 = quintile 4, 4 = quintile 5), food insecurity at each follow-up visit (none vs. any), parity (multiparous vs. nulliparous), and infant sex (female vs. male). *P*-values that are significant (<0.05) for each covariate are denoted by blue asterisk. Red asterisks denote that the *P*-value for the interaction term between time (study visit) and HIV exposure status is significant for that outcome. The *P*-values for the interaction term are LAZ (0.223), WAZ (0.388), WLZ (0.001), HCZ (0.009), and MUACZ (0.054). Limiting the above analysis to women on DTG-based ART vs. CHU, the P-values for the interaction terms are LAZ (0.272), WAZ (0.173), WLZ (<0.001), HCZ (0.038), and MUACZ (0.027). Full list of coefficients is presented in S4 Table. CHEU: Children HIV exposed uninfected; CHU, children HIV unexposed; LAZ, length-for-age Z-score; WAZ, weight-for-age Z-score; WLZ, weight-for-length Z-score; HCZ, head-circumference-for-age Z-score; MUAC, mid-upper arm circumference; MUACZ, MUAC-for-age Z-score.

CHEU had a greater mean WLZ than CHU at 1–7 days of birth (CHEU = −0.18 versus CHU = −0.37) with a decline in mean WLZ between week-6 and month-9 visits, and a steady increase between month-9 and month-24 visits (Table 2 and Fig 1). WLZ for CHU followed a similar pattern but remained greater than that of CHEU from week-6 onwards and stayed above the population average for all follow-up visits except the first. CHEU had significantly lower mean WLZ than CHU at 3-, 6-, and 9- months, but the findings were not significant in adjusted analysis. However, the overall trajectory for WLZ in the first 24 months was significantly lower for CHEU than CHU (interaction *p* = 0.001). By 24 months, 2.4%, and 7.1% of CHEU were wasted or overweight, compared to 0.6% and 6.4% of CHU, respectively (Fig 3 and S2 Table). CHEU had significantly greater risk of being wasted at month-12 than CHU (aRR: 3.60 [95% CI: 1.21, 10.70]).

Mean HCZ increased from birth to week-6 for both groups, but there was a greater change among CHU than CHEU (Table 2 and Fig 1). There was no statistically significant difference in mean HCZ or microcephaly or macrocephaly prevalence between the two groups at all visits except week-6 where CHU had significantly greater HCZ than CHEU. In adjusted regression analysis, the overall HCZ growth trajectory in the first two years was significantly slower for CHEU than CHU (interaction *p* = 0.009).

There was a modest increase in mean MUACZ over time for CHU and only after month-6 for CHEU. CHEU had significantly lower mean MUACZ than CHU at months 6, 9, 12, and 18, but the findings were no longer significant in the adjusted model. The overall trajectory of MUACZ growth in the first two years was not significantly different between the two groups (interaction *p* = 0.054).

Finally, among CHEU only, maternal ART initiation before versus during pregnancy was not associated with any difference in growth (all adjusted interactions *p* > 0.05) (S3 Table). In a sensitivity analysis limited to women on DTG-based ART (*n* = 154), the results of main analysis for the trajectory of LAZ, WAZ, WLZ, and HCZ comparing CHEU with CHU remained unchanged and in addition to WLZ and HCZ trajectories, the trajectory for MUACZ was lower among CHEU with DTG-based maternal ART than CHU during first two years (interaction *p* = 0.027). The results remained unchanged when comparing association between maternal ART initiation before pregnancy with all outcomes, among women on DTG-based ART.

Results of mixed effects model comparing CHEU with CHU for each growth outcome and adjusted for maternal age, any breastfeeding at each visit, education, depression, anemia, wealth index, food insecurity at each visit, parity, and child sex, revealed several cofactors that influenced child growth (Fig 3 and S4 Table). Breastfeeding and higher maternal education was associated with greater increase in growth trajectory in LAZ, WAZ, HCZ, and MUACZ in the first two years of life. Higher household wealth index positively influenced child’s LAZ and MUACZ growth. Male children had significantly lower LAZ, WAZ, and MUACZ trajectories than female children. There was no evidence that having at least one child at enrollment, food insecurity, and anemia during pregnancy impacted child growth.

## Discussion

This is one of the few studies comparing growth between CHU and CHEU in the context of WHO preferred DTG-based ART during pregnancy and breastfeeding among WLWH. Our findings show that CHEU had significantly lower WLZ and HCZ trajectories in the first two years of life, with increased risk of being underweight between months 9 and 24 than CHU. The prevalence of stunting tended to increase with age in this population, and by 24 months, nearly one-third of both CHEU and CHU were stunted.

We showed lower LAZ at birth, 18, and 24 months in CHEU than CHU, consistent with findings from a large Kenyan cohort in which nearly two-thirds of WLWH were on DTG-based ART in pregnancy, and lower LAZ was observed in CHEU at 6- and 12-months than CHU [36]. However, in the adjusted analysis in our study, CHEU and CHU demonstrated similar declines in linear growth trajectories from birth to two years. Although CHEU had lower LAZ at birth, their subsequent LAZ trajectory closely paralleled that of CHU, suggesting limited potential for catch-up growth in this cohort. These findings highlight the importance of growth monitoring and promotion, including nutritional education, complementary feeding support, and counseling, as critical strategies to enhance fetal growth, support linear growth among CHEU, and prevent stunting.

A recent study in South Africa, with shorter breastfeeding duration than that observed in our cohort, reported that breastfed CHEU with in utero efavirenz-based ART exposure had lower LAZ from 6–12 months and almost three times greater stunting risk at 12 months than CHU [16]. Shorter breastfeeding duration has been associated with more pronounced growth faltering in both CHEU and CHU in Bostwana [37]. In rural Zimbabwe, WLWH on non-DTG based ART regimens, and both CHEU and CHU breastfed for longer duration (>14 months), observed lower LAZ and higher stunting in CHEU (51%) than CHU (34%) at 18 months of age [13]. Another study in Zambia and South Africa, where most WLWH were on efavirenz + lamivudine + tenofovir ART regimen reported a difference in LAZ at 6 months despite no initial difference at 6–10 weeks between CHEU and CHU [38]. Notably, 72% of children were exclusively breastfeeding at 6–10 weeks which decreased to 38% at 6 months, with a lower proportion of exclusively breastfed CHEU than CHU [38]. We were unable to determine whether the linear growth observed between CHEU and CHU in our cohort as opposed to other studies is attributed to maternal DTG-based ART, longer breastfeeding duration, or other underlying pathophysiological differences. Further, although breastfeeding supports growth among CHEU and CHU, there is possibility of reverse causation such that poorly growing infants may be weaned early and rapidly growing infants and perceived healthy infants may breastfeed for a longer duration [39].

We showed a similar two-year weight trajectory between CHEU and CHU, but we observed that CHEU were more likely to be underweight from 9–24 months than CHU. This aligns with a study that reported no difference in WAZ at 6- and 12-months [36]. Our finding of increased risk of underweight among CHEU was similar to findings in Zimbabwe [13] and Kenya [36] that reported CHEU were more likely to be underweight at 6 months and 18 months than CHU. Contrary to our findings, CHEU in Zimbabwe however had lower WAZ at 18 months. Pooled data from Zambia and South Africa show that CHEU had lower WAZ at 6–10 weeks, but they exhibited weight catch-up growth by 6 months and the difference was no longer present [38]. Our findings of increasing overweight prevalence at 24 months among both CHEU (7.1%) and CHU (6.4%) highlight increased risk of early onset adult obesity and adverse metabolic health among these children. CHEU may be at risk of dyslipidemia and metabolic changes [40], and it is unknown whether obesity related metabolic changes are worsened by exposure to HIV and prenatal ART.

Our findings show some difference in proportional growth and head growth trajectories between CHEU and CHU. Despite no significant difference in wasting, microcephaly, or macrocephaly, the slower WLZ and HCZ growth trajectories in the first 2-years among CHEU in our cohort, compared to CHU, is noteworthy. Recent studies with similar wasting prevalence have shown no difference in WLZ at 6 months [36,38], 12 months [36], or 18 months [13]. In rural Zimbabwe, CHEU were shown to have lower HCZ and significant difference in microcephaly than CHU (10% versus 6%, respectively) [13]. However, the clinical value of slower HCZ change among CHEU in our study is unknown given that HCZ remained close to zero for both CHEU and CHU during the first two-years, with only a small proportion of micro- or macrocephaly.

Among CHEU, prepregnancy maternal ART was not associated with differences in two-year child growth outcomes. Limited studies have explored similar differences in low- and middle-income countries (LMICs). Similarly, a study in Kenya showed no significant associations of initiating ART during pregnancy or ART regimen with growth among CHEU [36]. Conversely, a clinical trial in Ethiopia found that exposure to maternal efavirenz- or protease inhibitor-based ART from conception, compared to later in pregnancy improved growth among CHEU [12]. A study conducted in a high-resource setting found that prepregnancy maternal ART was protective against infectious morbidity in CHEU [41]. The effects of in utero ART exposure may stem from physiological and metabolic adaptations, potentially mediated by epigenetic mechanisms with lasting implications for child growth. These gaps highlight the need for mechanistic research to elucidate these pathways and to inform the selection of the safest ARV regimens during pregnancy. Studies have reported that other HIV-related factors such as maternal viral load and immune activation (predicted by CD4 count) may influence child growth, although the findings are mixed [42–44]. Most women are virally suppressed in the current era of universal ART. Maternal viral suppression is associated with improved maternal health and decreased infant exposure to HIV and related opportunistic infections through breastmilk [11,45]. In our cohort, 91% of WLWH were virally suppressed during pregnancy, which could be an important predictor of improved growth among CHEU in our cohort [39]. Similar to a few other studies [36], we did not have data on maternal CD4 count and post-ART CD4 monitoring is no longer routinely measured in HIV-treatment programs in LMICs. Further work is needed to explore these findings with better characterization of maternal HIV related factors, for growth outcomes among CHEU in LMIC [41].

Consistent with previous studies [20,46,47], we found that male infants had lower LAZ, WAZ, and MUACZ. The biological mechanism behind this difference (and higher mortality and morbidity prevalence) is unclear although some may be attributed to differences in parenting and resource allocation, and sex hormone differences [46]. As expected, our findings show that higher maternal education and breastfeeding was associated with improved growth outcomes, which is above and beyond the impact of HIV exposure on growth. These findings highlight that providing support for breastfeeding for all children and continuing to promote sustained breastfeeding among CHEU in resource-limited settings has important considerations for improving child growth outcomes.

This study has several strengths and limitations. Breastfeeding support and infant nutrition counseling was provided to mothers in the study at each study visit which may have positively influenced growth outcomes. Additionally, mothers and children with moderate and severe acute malnutrition were referred to the nutritionist at the facility and provided nutritional supplementation per Kenyan guidelines. Although z-scores are commonly used in clinical practice to assess and monitor children’s growth and nutritional status, the significantly lower WAZ at 3, 6, 9, and 18 months and LAZ at birth, 18, and 24 months in CHEU compared to CHU may not represent clinically meaningful differences in weight or length. For instance, at the 18-month visit, the mean length and weight among male CHEU was 77.8 cm (SD 3.3) and 10.0 kg (SD 1.2), respectively, compared to 78.4 cm (SD 3.2) and 10.3 kg (SD 1.3) in male CHU. Among females, mean length and weight were 76.8 cm (SD 2.9) and 9.8 kg (SD 1.1) in CHEU, compared to 78.0 cm (SD 3.5) and 10.1 kg (SD 1.4) in CHU. These relatively small absolute differences suggest that, while statistically significant, their clinical relevance warrants careful interpretation. Interpreting multiple cofactors within a single model may increase the risk of a Table 2 fallacy in the results of our exploratory cofactor analysis. Findings from our study may not be generalizable to other settings with different characteristics (e.g., differing malnutrition prevalence, ART uptake and adherence). Finally, we could not account for biological pathways, including environmental enteric dysfunction and systemic inflammation, which have been shown to negatively impact growth and may be more marked in CHEU. Despite these limitations, this is a study of well-characterized CHEU with a CHU comparison group from the same community. We had high rates of follow-up of children throughout 24-month follow-up, and we conducted longitudinal HIV testing of children to avoid misclassification. Frequent follow-up visits and anthropometric measurements in duplicate by trained study staff make our findings robust. The DTG-based regimen used by WLWH in this cohort reflects the WHO preferred first-line treatment. There was high rate of viral suppression and breastfeeding in our cohort, reflecting the current and optimal scenario for prevention of vertical HIV transmission.

In summary, our study highlights growth deficits among breastfed CHEU, particularly poorer weight gain and slower weight-for-length growth. Slowed growth during the first two years of life is associated with adverse outcomes, including impaired cognitive development and lower educational attainment [48]. While early detection of growth deficits allows for timely interventions to prevent growth faltering, it is also important to recognize that rapid weight gain or catch-up growth in early childhood may predispose CHEU to adverse metabolic outcomes later in life [49]. Long-term monitoring is crucial to evaluate the impact of slowed early growth on CHEU as they transition into puberty and adolescence. Moreover, although CHEU evade HIV infection, the long-term consequences of ART exposure and the role of safer, sustained breastfeeding on CHEU growth and health outcomes remains poorly defined. Continued monitoring of growth of CHEU is critical to identify health disparities and future disease risks as HIV guidelines evolve, including changes in feeding recommendations, ART recommendations before and after pregnancy, and guidance on child ARV prophylaxis to prevent vertical transmission of HIV during breastfeeding. Further characterization of growth among CHEU in association with ART regimen, maternal nutrition, and breastfeeding duration is needed to implement strategies to improve growth of CHEU and optimize their health. A comprehensive approach that ensures universal access to ART, high-quality care, and sustained breastfeeding support for WLWH is essential to optimizing outcomes for CHEU. In parallel, well-designed mechanistic studies are needed to identify modifiable biological pathways underlying growth differences and to inform targeted interventions.

## Supporting information

S1 Inclusivity Checklist Inclusivity in global research. (DOCX)

S1 STROBE Checklist STrengthening the Reporting of OBservational studies in Epidemiology (STROBE) Statement—checklist of items that should be included in reports of observational studies, available at https://www.strobe-statement.org/, licenced under CC BY 4.0. (DOCX)

S1 Fig Flowchart of children in the Tunza Mwana cohort included in the growth analysis. *Outside catchment area includes residing outside catchment area and not remaining in the study area for 2 years. ^**^Not willing to participate includes not willing to return for follow-up, not willing to be contacted for follow-up visits or have home follow-up visits, not interested in participating, and not willing to provide informed consent.
(DOCX)

S1 Table Description of variables included in the multivariable regression models of association of HIV exposure on child growth outcomes. (DOCX)

S2 Table Child growth by visit among children HIV exposed uninfected and children HIV uninfected. ^a^The total number of children included in above calculations for each follow-up visits were 1–7 days of birth: 333 (CHEU: 171, CHU: 162); Week 3 visit: 315 (CHEU: 166, CHU: 149); Week 6 visit: 328 (CHEU: 169, CHU: 159); Month 3 visit: 329 (CHEU: 168, CHU: 161); Month 6 visit: 328 (CHEU: 167, CHU: 161); Month 9 visit: 326 (CHEU: 166, CHU: 160); Month 12 visit: 326 (CHEU: 166, CHU: 160); Month 18 visit: 326 (CHEU: 165, CHU: 161); Month 24 visit: 323 (CHEU: 165, CHU: 158). *p < 0.05 based on chi-squared test comparing CHEU and CHU for that visit. CHEU: Children HIV exposed uninfected; CHU: Children HIV unexposed uninfected; LAZ: Length-for-age Z-score; WAZ: Weight-for-age Z-score; WLZ: Weight-for-length Z-score; HCZ: Head-circumference-for-age Z-score; MUAC: Mid-upper arm circumference; MUACZ: MUAC-for-age Z-score.
(DOCX)

S3 Table Differences in growth trajectory by maternal ART initiation before pregnancy among CHEU over time using adjusted mixed effects linear regression model. *p < 0.05. β (95% CI) are difference in Z-scores between the groups specified, obtained from mixed-effects regression model with an interaction term between time (study visit) and the group. *P*-value are the values of interaction term between time (study visit) and the group for each outcome. Adjusted regression model additionally includes the following variables: maternal age (years), currently breastfeeding at each follow-up visit (days), education (Secondary and above vs. Primary or below), depression (yes/no), anemia (yes/no), wealth index (grouped linear variable – 0 = lowest quintile, 1 = quintile 2, 2 = quintile 3, 3 = quintile 4, 4 = quintile 5), food insecurity at each follow-up visit (secured vs. not secured), parity (multiparous vs. nulliparous), and infant sex (female vs male). CHEU, HIV exposed uninfected; CI, confidence interval; LAZ, length-for-age Z-score; WAZ, weight-for-age Z-score; WLZ, weight-for-length Z-score; HCZ, head-circumference-for-age Z-score; MUAC, mid-upper arm circumference; MUACZ, MUAC-for-age Z-score; NA, not applicable.
(DOCX)

S4 Table Coefficients of variables from multivariable mixed effects linear regression model. *p < 0.05. Values are mean difference (95% confidence interval); Multivariable regression model includes the following variables: maternal age (years), currently breastfeeding at each follow-up visit (days), education (Secondary and above vs. Primary or below), depression (yes/no), anemia (yes/no), wealth index (grouped linear variable – 0 = lowest quintile, 1 = quintile 2, 2 = quintile 3, 3 = quintile 4, 4 = quintile 5), food insecurity at each follow-up visit (secured vs. not secured), parity (multiparous vs. nulliparous), and infant sex (female vs. male), and interaction term between time (study visit) and HIV exposure status. CHEU, HIV exposed uninfected; LAZ, length-for-age Z-score; WAZ, weight-for-age Z-score; WLZ, weight-for-length Z-score; HCZ, head-circumference-for-age Z-score; MUAC, mid-upper arm circumference; MUACZ, MUAC-for-age Z-score.
(DOCX)

## Abbreviations

**:**
ART: antiretroviral therapy
ARV: antiretroviral
BMI: body mass index
CHEU: children who are HIV-exposed and uninfected
CHU: children who are HIV-unexposed
DTG: dolutegravir
HCZ: head circumference-for-age Z-scores
IQR: interquartile range
LAZ: length-for-age Z-scores
LMICs: low- and middle-income countries
MCH: Maternal and Child Health
MUAC: mid-upper arm circumference-for-age Z-scores
PHQ-9: Patient Health Questionnaire-9
SD: standard deviation
STROBE: Strengthening the Reporting of Observational Studies in Epidemiology
WAZ: weight-for-age Z-scores
WLWH: women living with HIV
WLZ: weight-for-length Z-scores
