# Supplementary material for: Differences in growth trajectories in breastfed HIV-exposed uninfected and HIV-unexposed infants in Kenya: An observational cohort study
Source: PLoS Med. 2025 Oct 27;22(10):e1004781. doi: 10.1371/journal.pmed.1004781 (PMC12578329; doi:10.1371/journal.pmed.1004781)
Supplement: S1 Fig — *Outside catchment area includes residing outside catchment area and not remaining in the study area for 2 years. **Not willing to participate includes not willing to return for follow-up, not willing to be contacted for follow-up visits or have home follow-up visits, not interested in participating, and not willing to provide informed consent. (DOCX) [file pmed.1004781.s003.docx]

843 excluded

563 were <28 or >42 weeks’ gestation

250 not willing to participate^**^

133 outside catchment area^*^

91 aged <18 and >40 years

23 previously enrolled

12 preeclampsia/hypertension

11 not planning to breastfeed

7 unknown HIV status

3 other

1442 pregnant women screened for eligibility

350 pregnant women enrolled

175 pregnant women living with HIV

175 pregnant women without HIV

13 excluded (<2 growth measures)

7 voluntary withdrawals

2 child deaths

1 fetal death

1 congenital anomaly

1 untraceable

1 women HIV positive at delivery

4 excluded (<2 growth measures)

2 voluntary withdrawals

1 child death

1 moved out of area

171 children HIV-exposed uninfected included in growth analysis

162 children HIV unexposed included in growth analysis

**S1 Fig. Flowchart of children in the Tunza Mwana cohort included in the growth analysis.**
