# Supplementary material for: Differences in growth trajectories in breastfed HIV-exposed uninfected and HIV-unexposed infants in Kenya: An observational cohort study
Source: PLoS Med. 2025 Oct 27;22(10):e1004781. doi: 10.1371/journal.pmed.1004781 (PMC12578329; doi:10.1371/journal.pmed.1004781)
Supplement: S1 Table — (DOCX) [file pmed.1004781.s004.docx]

**S1 Table. Description of variables included in the multivariable regression models of association of HIV exposure on child growth outcomes**

| **Variables** | **Description** |
| --- | --- |
| HIV exposure status | HIV test result of the pregnant women |
| Study visit | - 1-7 days of birth - Week 3 visit - Week 6 visit - Month 3 visit - Month 6 visit - Month 9 visit - Month 12 visit - Month 18 visit - Month 24 visit |
| Maternal age *(years)* | Age of the pregnant women (in years, range between ≥18 years and ≤40 years) at enrolment |
| Time-varying breastfeeding *(yes, no)* | At each follow-up visit, the following question was asked to the mothers: Are you currently breastfeeding your infant? |
| Maternal education *(Secondary and above, Primary or below)* | What is your highest level of education?   - No formal education - Less than primary - Primary completed - Secondary – not completed - Secondary – completed - Above secondary   No formal education, Less than primary, Primary completed, and Secondary – not completed were combined to denote *Primary or below.*  Secondary – completed and Above secondary was combined to denote *Secondary and above.* |
| Depression (yes, no) | Overall scores from Patient Health Questionnaire (PHQ-9) scale were categorized as follows for each participant:   - No to minimal depression: <5 - Mild depression: >=5 & <10 - Moderate or severe depression: >= 10   No to minimal depression was used to denote *No* depression and Mild or moderate or severe depression was used to denote depression. |
| Anemia during pregnancy (yes, no) | Hemoglobin count < 11 mg/dL at enrolment |
| Household wealth index* (lowest quintile, quintile 2, quintile 3, quintile 4, quintile 5) | A score that represented each child was derived using principal component analysis from the following information for each child.   - Do you or a member of your household have a bank account? - What is the main source of fuel for cooking in your house?   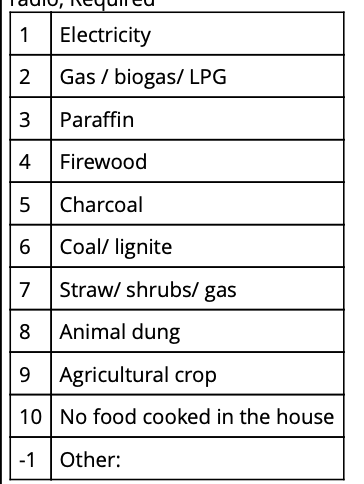   - What type of toilet do you have?   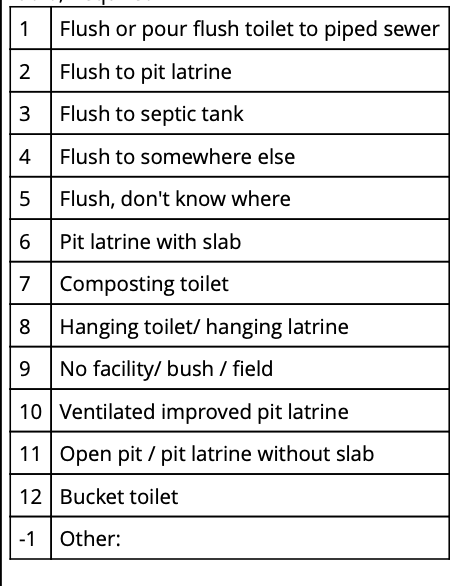   - What is the main source of drinking water for members of your   household?  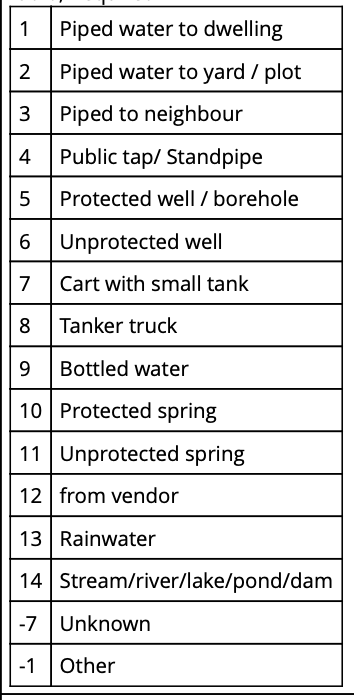   - What is the MAIN floor material of the rooms in your household?   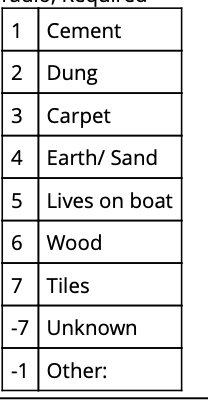   - What is the MAIN wall material in your household?   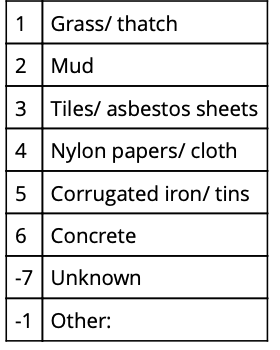   - What is the MAIN roof material of the house in this household?   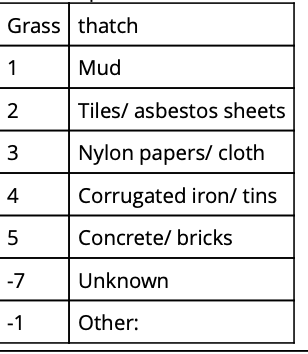   - Does your household have a radio? - Does your household have Electricity? - Does your household have A television? - Does your household have A refrigerator? - Does your household have A bicycle? - A Does your household have motorcycle scooter? - Does any member of your household have a mobile phone?   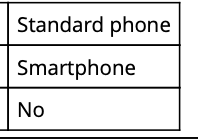   - Does this household have any of the following animals? Horses, cows, goats, sheep, chicken, ducks, or pigs   Improved drinking water [1], improved cooking [2], improved floor[3], improved roof[3], improved wall[3], improved sanitation [1,4] were further determined from the above information.  These scores were split into quintiles and a child classified within the highest quintile or quintile 5 indicates greater household wealth |
| Time-varying household food insecurity *(none, any)* | Categorized as per Household Food Insecurity Access Scale (HFIAS) for Measurement of Household Food Access for each follow-up visit.   - Secured - Mild insecurity - Moderate insecurity - Severe insecurity   Secured was used to denote “none” insecurity and mild, moderate, or severe categories were used to denote *any* food insecurity |
| Parity at enrolment *(multiparous, nulliparous)* | Total number of pregnancies (including current) |
| Infant sex *(male, female)* | What was/is the sex of your baby (first baby' if multiple babies)? |

**References**

1. Rakotomanana H, Komakech JJ, Walters CN, Stoecker BJ. The WHO and UNICEF Joint Monitoring Programme (JMP) Indicators for Water Supply, Sanitation and Hygiene and Their Association with Linear Growth in Children 6 to 23 Months in East Africa. Int J Environ Res Public Health. 2020;17: 6262. doi:10.3390/ijerph17176262

2. Clean and Improved Cooking in Sub-Saharan Africa November 2014 Second Edition. World Bank Group; 2014. Available: https://openknowledge.worldbank.org/bitstream/handle/10986/22521/Clean0and0impr000a0landscape0report.pdf?sequence=1&isAllowed=y

3. Florey L, Taylor C. Using household survey data to explore the effects of improved housing conditions on malaria infection in children in Sub-Saharan Africa. Rockville, Maryland, USA: ICF International; 2016. Available: http://dhsprogram.com/pubs/pdf/AS61/AS61.pdf

4. World Health Organization. Improved sanitation facilities and drinking-water sources. [cited 30 Jan 2023]. Available: https://www.who.int/data/nutrition/nlis/info/improved-sanitation-facilities-and-drinking-water-sources
