# Supplementary material for: Differences in growth trajectories in breastfed HIV-exposed uninfected and HIV-unexposed infants in Kenya: An observational cohort study
Source: PLoS Med. 2025 Oct 27;22(10):e1004781. doi: 10.1371/journal.pmed.1004781 (PMC12578329; doi:10.1371/journal.pmed.1004781)
Supplement: S2 Table — aThe total number of children included in above calculations for each follow-up visits were 1–7 days of birth: 333 (CHEU: 171, CHU: 162); Week 3 visit: 315 (CHEU: 166, CHU: 149); Week 6 visit: 328 (CHEU: 169, CHU: 159); Month 3 visit: 329 (CHEU: 168, CHU: 161); Month 6 visit: 328 (CHEU: 167, CHU: 161); Month 9 visit: 326 (CHEU: 166, CHU: 160); Month 12 visit: 326 (CHEU: 166, CHU: 160); Month 18 visit: 326 (CHEU: 165, CHU: 161); Month 24 visit: 323 (CHEU: 165, CHU: 158). *p < 0.05 based on chi-squared test comparing CHEU and CHU for that visit. CHEU: Children HIV exposed uninfected; CHU: Children HIV unexposed uninfected; LAZ: Length-for-age Z-score; WAZ: Weight-for-age Z-score; WLZ: Weight-for-length Z-score; HCZ: Head-circumference-for-age Z-score; MUAC: Mid-upper arm circumference; MUACZ: MUAC-for-age Z-score. (DOCX) [file pmed.1004781.s005.docx]

**S2 Table. Child growth by visit among children HIV exposed uninfected and children HIV uninfected^a^**

|  | **Stunting (LAZ<-2)** | **Underweight**  **(WAZ<-2)** | **Wasting**  **(WLZ<-2)** | **Overweight (WLZ>2)** | **Micro-cephaly (HCZ<-2)** | **Macro-cephaly (HCZ>2)** | **Stunted and wasted** | **Overweight and**  **stunted** |
| --- | --- | --- | --- | --- | --- | --- | --- | --- |
| ***Children HIV exposed uninfected (CHEU)*** | | | | | | | | |
| 1-7 days of birth | 16 (9.4%) | 9 (5.3%) | 9 (5.3%) | 5 (2.9%) | 8 (4.7%) | 5 (2.9%) | 0 (0%) | 0 (0%) |
| Week 3 visit | 15 (9.0%) | 9 (5.4%) | 6 (3.6%) | 9 (5.3%) | 4 (2.3%) | 7 (4.1%) | 1 (0.6%) | 2 (1.2%) |
| Week 6 visit | 12 (7.1%) | 9 (5.3%) | 3 (1.8%) | 15 (8.8%) | 3 (1.8%) | 6 (3.5%) | 1 (0.6%) | 4 (2.4%) |
| Month 3 visit | 13 (7.7%) | 13 (7.7%) | 9 (5.4%) | 17 (9.9%) | 6 (3.5%) | 8 (4.7%) | 2 (1.2%) | 4 (2.4%) |
| Month 6 visit | 20 (12.0%) | 14 (8.4%) | 6 (3.6%) | 9 (5.3%) | 9 (5.3%) | 1 (0.6%) | 0 (0%) | 1 (0.6%) |
| Month 9 visit | 21 (12.7%) | 17 (10.2%) | 9 (5.4%) | 5 (3.0%) | 6 (3.6%) | 3 (1.8%) | 3 (1.8%) | 0 (0.0%) |
| Month 12 visit | 25 (15.1%) | 21 (12.7%) | 7 (4.2%) | 5 (3.0%) | 6 (3.6%) | 3 (1.8%) | 4 (2.4%) | 0 (0.0%) |
| Month 18 visit | 46 (27.9%) | 17 (10.3%) | 5 (3.0%) | 5 (3.0%) | 4 (2.4%) | 4 (2.4%) | 4 (2.4%) | 2 (1.2%) |
| Month 24 visit | 52 (31.5%) | 16 (9.3%) | 4 (2.4%) | 12 (7.1%) | 7 (4.1%) | 3 (1.8%) | 2 (1.2%) | 4 (2.5%) |
| ***Children HIV unexposed (CHU)*** | | | | | | | | |
| 1-7 days of birth | 12 (7.4%) | 11 (6.8%) | 14 (8.6%) | 5 (3.1%) | 5 (3.1%) | 4 (2.5%) | 2 (1.2%) | 1 (0.6%) |
| Week 3 visit | 9 (6.0%) | 6 (4.0%) | 7 (4.7%) | 5 (3.4%) | 5 (3.4%) | 7 (4.7%) | 2 (1.3%) | 0 (0.0%) |
| Week 6 visit | 13 (8.2%) | 4 (2.5%) | 4 (2.5%) | 13 (8.2%) | 8 (5.0%) | 10 (6.3%) | 1 (0.6%) | 3 (1.9%) |
| Month 3 visit | 20 (12.4%) | 7 (4.3%) | 2 (1.2%) | 21 (13.0%) | 4 (2.5%) | 6 (3.7%) | 0 (0%) | 7 (4.3%) |
| Month 6 visit | 16 (9.9%) | 9 (5.6%) | 3 (1.9%) | 17 (10.6%) | 5 (3.1%) | 2 (1.2%) | 0 (0%) | 6 (3.7%) |
| Month 9 visit | 13 (8.1%) | 6 (3.8%) | 4 (2.5%) | 10 (6.3%) | 5 (3.1%) | 1 (0.6%) | 1 (0.6%) | 2 (1.3%) |
| Month 12 visit | 24 (15.0%) | 7 (4.4%) | 2 (1.3%) | 7 (4.4%) | 4 (2.5%) | 2 (1.3%) | 0 (0%) | 0 (0.0%) |
| Month 18 visit | 40 (24.8%) | 7 (4.3%) | 2 (1.2%) | 7 (4.3%) | 4 (2.5%) | 1 (0.6%) | 1 (0.6%) | 0 (0.0%) |
| Month 24 visit | 43 (27.2%) | 5 (3.2%) | 1 (0.6%) | 10 (6.4%) | 1 (0.6%) | 4 (2.5%) | 0 (0%) | 2 (1.3%) |
