# Supplementary material for: Differences in growth trajectories in breastfed HIV-exposed uninfected and HIV-unexposed infants in Kenya: An observational cohort study
Source: PLoS Med. 2025 Oct 27;22(10):e1004781. doi: 10.1371/journal.pmed.1004781 (PMC12578329; doi:10.1371/journal.pmed.1004781)
Supplement: S3 Table — *p < 0.05. β (95% CI) are difference in Z-scores between the groups specified, obtained from mixed-effects regression model with an interaction term between time (study visit) and the group. P-value are the values of interaction term between time (study visit) and the group for each outcome. Adjusted regression model additionally includes the following variables: maternal age (years), currently breastfeeding at each follow-up visit (days), education (Secondary and above vs. Primary or below), depression (yes/no), anemia (yes/no), wealth index (grouped linear variable – 0 = lowest quintile, 1 = quintile 2, 2 = quintile 3, 3 = quintile 4, 4 = quintile 5), food insecurity at each follow-up visit (secured vs. not secured), parity (multiparous vs. nulliparous), and infant sex (female vs male). CHEU, HIV exposed uninfected; CI, confidence interval; LAZ, length-for-age Z-score; WAZ, weight-for-age Z-score; WLZ, weight-for-length Z-score; HCZ, head-circumference-for-age Z-score; MUAC, mid-upper arm circumference; MUACZ, MUAC-for-age Z-score; NA, not applicable. (DOCX) [file pmed.1004781.s006.docx]

**S3 Table: Differences in growth trajectory by maternal ART initiation before pregnancy among CHEU over time using adjusted mixed effects linear regression model**

|  | **β (95% CI)** | | | | |
| --- | --- | --- | --- | --- | --- |
|  | **LAZ** | **WAZ** | **WLZ** | **HCZ** | **MUACZ** |
| ***Crude regression model*** | | | | | |
| 1-7 days of birth | -0.17 (-0.68, 0.34) | -0.02 (-0.54, 0.5) | 0.15 (-0.45, 0.75) | -0.20 (-0.7, 0.30) | NA |
| Week 3 visit | -0.19 (-0.7, 0.33) | -0.07 (-0.6, 0.45) | 0.13 (-0.46, 0.73) | -0.18 (-0.69, 0.32) | NA |
| Week 6 visit | -0.11 (-0.62, 0.40) | -0.09 (-0.61, 0.44) | -0.02 (-0.61, 0.58) | -0.26 (-0.76, 0.25) | NA |
| Month 3 visit | 0.05 (-0.47, 0.56) | -0.11 (-0.63, 0.41) | -0.20 (-0.8, 0.39) | -0.21 (-0.72, 0.29) | -0.13 (-0.63, 0.37) |
| Month 6 visit | -0.25 (-0.77, 0.27) | -0.36 (-0.89, 0.16) | -0.27 (-0.87, 0.33) | -0.24 (-0.74, 0.27) | -0.21 (-0.66, 0.23) |
| Month 9 visit | -0.14 (-0.65, 0.38) | -0.31 (-0.83, 0.22) | -0.32 (-0.91, 0.28) | -0.40 (-0.9, 0.11) | -0.26 (-0.71, 0.18) |
| Month 12 visit | -0.18 (-0.7, 0.33) | -0.38 (-0.9, 0.15) | -0.40 (-1.0, 0.2.0) | -0.42 (-0.93, 0.08) | -0.36 (-0.81, 0.08) |
| Month 18 visit | -0.18 (-0.70, 0.33) | -0.35 (-0.88, 0.17) | -0.37 (-0.97, 0.22) | -0.32 (-0.82, 0.19) | -0.33 (-0.77, 0.12) |
| Month 24 visit | -0.23 (-0.75, 0.28) | -0.38 (-0.9, 0.15) | -0.34 (-0.94, 0.27) | -0.21 (-0.72, 0.29) | -0.3 (-0.74, 0.14) |
| **Adjusted regression model** | | | | | |
| *P-value* | *0.725* | *0.373* | *0.668* | *0.846* | *0.904* |
| 1-7 days of birth | -0.17 (-0.70, 0.36) | 0.02 (-0.53, 0.58) | 0.15 (-0.49, 0.8) | -0.08 (-0.62, 0.45) | NA |
| Week 3 visit | -0.22 (-0.74, 0.31) | -0.09 (-0.64, 0.46) | 0.12 (-0.51, 0.75) | -0.13 (-0.66, 0.41) | NA |
| Week 6 visit | -0.17 (-0.7, 0.35) | -0.14 (-0.69, 0.41) | -0.05 (-0.69, 0.59) | -0.20 (-0.73, 0.34) | NA |
| Month 3 visit | -0.03 (-0.56, 0.49) | -0.15 (-0.71, 0.4) | -0.18 (-0.81, 0.46) | -0.19 (-0.72, 0.34) | -0.22 (-0.71, 0.28) |
| Month 6 visit | -0.41 (-0.93, 0.12) | -0.42 (-0.97, 0.14) | -0.20 (-0.83, 0.44) | -0.22 (-0.75, 0.32) | -0.28 (-0.72, 0.17) |
| Month 9 visit | -0.20 (-0.73, 0.32) | -0.26 (-0.81, 0.29) | -0.20 (-0.84, 0.43) | -0.35 (-0.88, 0.18) | -0.19 (-0.64, 0.25) |
| Month 12 visit | -0.24 (-0.77, 0.28) | -0.27 (-0.82, 0.28) | -0.22 (-0.85, 0.42) | -0.30 (-0.84, 0.23) | -0.27 (-0.71, 0.18) |
| Month 18 visit | -0.24 (-0.76, 0.29) | -0.28 (-0.83, 0.27) | -0.24 (-0.87, 0.40) | -0.23 (-0.76, 0.31) | -0.24 (-0.69, 0.20) |
| Month 24 visit | -0.28 (-0.8, 0.25) | -0.26 (-0.82, 0.29) | -0.15 (-0.80, 0.49) | -0.18 (-0.71, 0.36) | -0.13 (-0.58, 0.32) |
