# Supplementary material for: Differences in growth trajectories in breastfed HIV-exposed uninfected and HIV-unexposed infants in Kenya: An observational cohort study
Source: PLoS Med. 2025 Oct 27;22(10):e1004781. doi: 10.1371/journal.pmed.1004781 (PMC12578329; doi:10.1371/journal.pmed.1004781)
Supplement: S4 Table — *p < 0.05. Values are mean difference (95% confidence interval); Multivariable regression model includes the following variables: maternal age (years), currently breastfeeding at each follow-up visit (days), education (Secondary and above vs. Primary or below), depression (yes/no), anemia (yes/no), wealth index (grouped linear variable – 0 = lowest quintile, 1 = quintile 2, 2 = quintile 3, 3 = quintile 4, 4 = quintile 5), food insecurity at each follow-up visit (secured vs. not secured), parity (multiparous vs. nulliparous), and infant sex (female vs. male), and interaction term between time (study visit) and HIV exposure status. CHEU, HIV exposed uninfected; LAZ, length-for-age Z-score; WAZ, weight-for-age Z-score; WLZ, weight-for-length Z-score; HCZ, head-circumference-for-age Z-score; MUAC, mid-upper arm circumference; MUACZ, MUAC-for-age Z-score. (DOCX) [file pmed.1004781.s007.docx]

**S4 Table: Coefficients of variables from multivariable mixed effects linear regression model**

|  | **LAZ** | **WAZ** | **WLZ** | **HCZ** | **MUACZ** |
| --- | --- | --- | --- | --- | --- |
| ***Crude regression model*** |  |  |  |  |  |
| Intercept | -0.39 (-0.56, -0.22)* | -0.35 (-0.52, -0.18)* | -0.37 (-0.55, -0.18)* | 0.09 (-0.08, 0.25) | 0.31 (0.14, 0.49)* |
| CHEU vs CHU | -0.29 (-0.52, -0.05)* | -0.14 (-0.38, 0.09) | 0.18 (-0.08, 0.43)* | -0.19 (-0.41, 0.04) | -0.27 (-0.52, -0.02)* |
| Week 3 visit | 0.01 (-0.14, 0.15) | 0.21 (0.07, 0.35)* | 0.5 (0.31, 0.7)* | 0.05 (-0.08, 0.17) | - |
| Week 6 visit | -0.09 (-0.24, 0.05) | 0.28 (0.14, 0.42)* | 0.97 (0.78, 1.16)* | 0.13 (0.00, 0.26)* | - |
| Month 3 visit | -0.23 (-0.37, -0.09)* | 0.33 (0.19, 0.47)* | 1.06 (0.87, 1.25)* | 0.08 (-0.05, 0.21) | - |
| Month 6 visit | -0.2 (-0.34, -0.06)* | 0.26 (0.13, 0.4)* | 0.82 (0.63, 1.01)* | -0.19 (-0.32, -0.07)* | -0.03 (-0.17, 0.12) |
| Month 9 visit | -0.21 (-0.35, -0.07)* | 0.18 (0.04, 0.32)* | 0.62 (0.43, 0.82)* | -0.27 (-0.39, -0.14)* | 0.06 (-0.09, 0.2) |
| Month 12 visit | -0.45 (-0.59, -0.31)* | 0.08 (-0.06, 0.22) | 0.55 (0.36, 0.75) | -0.24 (-0.36, -0.11)* | 0.14 (0.00, 0.29) |
| Month 18 visit | -0.82 (-0.96, -0.68)* | -0.04 (-0.18, 0.1) | 0.6 (0.41, 0.79)* | -0.13 (-0.26, -0.01)* | 0.09 (-0.06, 0.23) |
| Month 24 visit | -0.9 (-1.04, -0.76)* | -0.01 (-0.15, 0.13) | 0.78 (0.59, 0.97)* | -0.04 (-0.16, 0.09) | -0.08 (-0.23, 0.06) |
| CHEU:visitweek-3 | 0.04 (-0.16, 0.24) | -0.06 (-0.26, 0.13) | -0.16 (-0.43, 0.11) | -0.04 (-0.22, 0.14) | - |
| CHEU:visitweek-6 | 0.22 (0.02, 0.41)* | -0.06 (-0.25, 0.13) | -0.39 (-0.66, -0.12)* | -0.1 (-0.27, 0.08) | - |
| CHEU:visitmonth-3 | 0.27 (0.08, 0.47)* | -0.15 (-0.34, 0.04) | -0.59 (-0.85, -0.32)* | 0.02 (-0.15, 0.2) | - |
| CHEU:visitmonth-6 | 0.14 (-0.06, 0.33) | -0.17 (-0.36, 0.02) | -0.46 (-0.73, -0.19)* | 0.09 (-0.09, 0.26) | -0.03 (-0.23, 0.17) |
| CHEU:visitmonth-9 | 0.15 (-0.05, 0.34) | -0.19 (-0.38, 0) | -0.51 (-0.78, -0.24)* | 0.16 (-0.02, 0.33) | -0.03 (-0.23, 0.17) |
| CHEU:visitmonth-12 | 0.17 (-0.03, 0.36) | -0.09 (-0.28, 0.1) | -0.41 (-0.68, -0.14)* | 0.20 (0.03, 0.38)* | 0.00 (-0.20, 0.20) |
| CHEU:visitmonth-18 | 0.00 (-0.2, 0.19) | -0.12 (-0.32, 0.07) | -0.34 (-0.61, -0.07)* | 0.23 (0.06, 0.41)* | -0.02 (-0.22, 0.18) |
| CHEU:visitweek-24 | 0.03 (-0.17, 0.23) | -0.03 (-0.22, 0.17) | -0.24 (-0.51, 0.03) | 0.02 (-0.16, 0.20) | 0.14 (-0.06, 0.35) |
| ***Adjusted regression model*** |  |  |  |  |  |
| Intercept | -1.13 (-1.70, -0.57)* | -0.66 (-1.24, -0.09)* | -0.12 (-0.69, 0.45) | -0.61 (-1.21, -0.08)* | 0.12 (-0.44, 0.67) |
| CHEU vs CHU | -0.18 (-0.44, 0.08) | -0.04 (-0.3, 0.22) | 0.21 (-0.08, 0.5) | -0.09 (-0.35, 0.17) | -0.06 (-0.33, 0.21) |
| Week 3 visit | 0.07 (-0.07, 0.22) | 0.23 (0.08, 0.37)* | 0.43 (0.22, 0.63)* | 0.09 (-0.04, 0.22) | - |
| Week 6 visit | -0.02 (-0.16, 0.13) | 0.3 (0.16, 0.43)* | 0.88 (0.69, 1.08)* | 0.17 (0.04, 0.3)* | - |
| Month 3 visit | -0.16 (-0.30, -0.02)* | 0.34 (0.2, 0.48)* | 0.97 (0.77, 1.17)* | 0.12 (-0.01, 0.25) | - |
| Month 6 visit | -0.09 (-0.24, 0.05) | 0.28 (0.14, 0.42)* | 0.72 (0.52, 0.91)* | -0.14 (-0.26, -0.01)* | -0.04 (-0.18, 0.1) |
| Month 9 visit | -0.11 (-0.25, 0.03) | 0.2 (0.06, 0.34)* | 0.53 (0.33, 0.73)* | -0.21 (-0.34, -0.08)* | 0.07 (-0.07, 0.21) |
| Month 12 visit | -0.33 (-0.48, -0.19)* | 0.12 (-0.02, 0.26) | 0.47 (0.27, 0.67)* | -0.16 (-0.29, -0.03)* | 0.17 (0.03, 0.31)* |
| Month 18 visit | -0.63 (-0.78, -0.49)* | 0.05 (-0.1, 0.19) | 0.52 (0.32, 0.72)* | -0.02 (-0.15, 0.11) | 0.16 (0.02, 0.31)* |
| Month 24 visit | -0.58 (-0.74, -0.42)* | 0.15 (-0.01, 0.31) | 0.71 (0.49, 0.93)* | 0.17 (0.02, 0.31)* | 0.06 (-0.09, 0.22) |
| Maternal age (years) | 0.00 (-0.02, 0.02) | -0.01 (-0.03, 0.01) | -0.01 (-0.03, 0.01) | 0 (-0.02, 0.02) | -0.01 (-0.03, 0.01) |
| Parity (multiparous vs. nulliparous) | 0.14 (-0.15, 0.44) | 0.23 (-0.07, 0.54) | 0.18 (-0.1, 0.47) | 0.23 (-0.07, 0.53) | 0.08 (-0.21, 0.36) |
| Any breastfeeding at each follow-up visit | 0.33 (0.22, 0.44)* | 0.2 (0.09, 0.31)* | 0.02 (-0.13, 0.17) | 0.21 (0.11, 0.31)* | 0.20 (0.11, 0.30)* |
| Education (Secondary and above vs. Primary or below) | 0.43 (0.20, 0.65)* | 0.28 (0.04, 0.51)* | 0.02 (-0.2, 0.25) | 0.31 (0.08, 0.55)* | 0.23 (0.01, 0.46)* |
| Depression (yes/no) | 0.15 (-0.07, 0.36) | 0.23 (0.01, 0.45)* | 0.17 (-0.03, 0.38) | 0.25 (0.03, 0.47)* | 0.15 (-0.06, 0.36)* |
| Anemia (yes/no) | 0.05 (-0.16, 0.27) | -0.09 (-0.32, 0.13) | -0.15 (-0.37, 0.06) | -0.08 (-0.31, 0.14) | -0.08 (-0.29, 0.14) |
| Wealth quintile (0=lowest quintile, 4=highest wealth quintile) | 0.06 (0.02, 0.13)* | 0.05 (-0.03, 0.13) | 0.02 (-0.06, 0.09) | 0.05 (-0.03, 0.12) | 0.07 (0.00, 0.15)* |
| Food insecurity (secure vs. not secure) at each follow-up visit | -0.02 (-0.05, 0.01) | 0.02 (-0.05, 0.08) | -0.01 (-0.03, 0.05) | 0.00 (-0.03, 0.02) | -0.01 (-0.04, 0.022) |
| Child sex (Male vs Female) | -0.26 (-0.46, -0.06)* | -0.26 (-0.47, -0.06)* | -0.13 (-0.32, 0.06) | -0.06 (-0.26, 0.14) | -0.23 (-0.42, -0.03)* |
| CHEU:visitweek-3 | 0.02 (-0.18, 0.23) | -0.06 (-0.26, 0.14) | -0.14 (-0.42, 0.15) | -0.05 (-0.24, 0.13) | - |
| CHEU:visitweek-6 | 0.17 (-0.03, 0.37) | -0.08 (-0.28, 0.12) | -0.36 (-0.64, -0.08)* | -0.13 (-0.31, 0.05) | - |
| CHEU:visitmonth-3 | 0.26 (0.06, 0.46)* | -0.16 (-0.36, 0.03) | -0.59 (-0.88, -0.31)* | -0.01 (-0.2, 0.17) | - |
| CHEU:visitmonth-6 | 0.08 (-0.12, 0.28) | -0.18 (-0.38, 0.01) | -0.43 (-0.71, -0.15)* | 0.03 (-0.16, 0.21) | -0.06 (-0.26, 0.14) |
| CHEU:visitmonth-9 | 0.11 (-0.09, 0.31) | -0.23 (-0.43, -0.03)* | -0.53 (-0.81, -0.25)* | 0.09 (-0.09, 0.27) | -0.10 (-0.29, 0.1) |
| CHEU:visitmonth-12 | 0.11 (-0.09, 0.32) | -0.13 (-0.33, 0.07) | -0.42 (-0.7, -0.14)* | 0.13 (-0.05, 0.31) | -0.08 (-0.27, 0.12) |
| CHEU:visitmonth-18 | 0.01 (-0.19, 0.22) | -0.11 (-0.31, 0.09) | -0.32 (-0.61, -0.04)* | 0.23 (0.04, 0.41)* | -0.01 (-0.21, 0.18) |
| CHEU:visitweek-24 | 0.06 (-0.14, 0.27) | -0.04 (-0.24, 0.17) | -0.27 (-0.55, 0.02) | 0.02 (-0.17, 0.21) | 0.13 (-0.07, 0.32) |
